# Time-resolved metabolomics reveals metabolic modulation in rice foliage

**DOI:** 10.1186/1752-0509-2-51

**Published:** 2008-06-18

**Authors:** Shigeru Sato, Masanori Arita, Tomoyoshi Soga, Takaaki Nishioka, Masaru Tomita

**Affiliations:** 1 Institute for Advanced Biosciences, Keio University, Tsuruoka, Japan; 2 Department of Computational Biology, Graduate School of Frontier Sciences, The University of Tokyo and PRESTO-JST, Kashiwa, Japan; 3 Plant Science Center, Riken, Yokohama, Japan; 4 Human Metabolome Technologies, Inc., Tsuruoka, Japan; 5 Graduate School of Agriculture, Kyoto University, Kyoto, Japan

## Abstract

**Background:**

To elucidate the interaction of dynamics among modules that constitute biological systems, comprehensive datasets obtained from "omics" technologies have been used. In recent plant metabolomics approaches, the reconstruction of metabolic correlation networks has been attempted using statistical techniques. However, the results were unsatisfactory and effective data-mining techniques that apply appropriate comprehensive datasets are needed.

**Results:**

Using capillary electrophoresis mass spectrometry (CE-MS) and capillary electrophoresis diode-array detection (CE-DAD), we analyzed the dynamic changes in the level of 56 basic metabolites in plant foliage (*Oryza sativa *L. ssp. *japonica*) at hourly intervals over a 24-hr period. Unsupervised clustering of comprehensive metabolic profiles using Kohonen's self-organizing map (SOM) allowed classification of the biochemical pathways activated by the light and dark cycle. The carbon and nitrogen (C/N) metabolism in both periods was also visualized as a phenotypic linkage map that connects network modules on the basis of traditional metabolic pathways rather than pairwise correlations among metabolites. The regulatory networks of C/N assimilation/dissimilation at each time point were consistent with previous works on plant metabolism. In response to environmental stress, glutathione and spermidine fluctuated synchronously with their regulatory targets. Adenine nucleosides and nicotinamide coenzymes were regulated by phosphorylation and dephosphorylation. We also demonstrated that SOM analysis was applicable to the estimation of unidentifiable metabolites in metabolome analysis. Hierarchical clustering of a correlation coefficient matrix could help identify the bottleneck enzymes that regulate metabolic networks.

**Conclusion:**

Our results showed that our SOM analysis with appropriate metabolic time-courses effectively revealed the synchronous dynamics among metabolic modules and elucidated the underlying biochemical functions. The application of discrimination of unidentified metabolites and the identification of bottleneck enzymatic steps even to non-targeted comprehensive analysis promise to facilitate an understanding of large-scale interactions among components in biological systems.

## Background

In the post-genome era, comprehensive data from "omics" technologies (genomics, transcriptomics, proteomics, and metabolomics) have been extensively analyzed to elucidate the underlying biochemical networks that elaborately regulate cellular mechanisms. Recent contributions from metabolomics are particularly noteworthy; they offer insights into metabolism that complement information obtained from proteomics and transcriptomics [1]. Correlation analysis of metabolic profiles has been used effectively to distinguish silent phenotypes or genetic alterations that are not noticeable superficially [2-4]. The systematic integration of metabolomic-, proteomic-, and transcriptomic profiles facilitates the unbiased, information-based reconstruction of underlying biochemical networks [5,6]. Kohonen's self-organizing map (SOM) analysis [7] was also an effective method to classify and monitor metabolic alteration patterns with time-series profiles [8,9].

However, with the current technology, unbiased reconstruction from comprehensive and high-throughput data is challenging; statistical tools are immature and inherent measurement errors and biological noise continue to present problems [10]. Moreover, two issues are relevant to the exploitation of metabolomics data. First, it is crucial to interpret metabolic profiles by focusing on a specific rhythm in an appropriate time range and interval, since plants have adapted their metabolism to different environmental fluctuations such as the slow and steady diurnal rhythm, whereas metabolic levels change dynamically. Second, currently available metabolomics data are insufficient for the detection of new metabolic networks. Even if non-target profiling were able to quantify thousands of metabolites, at present there is no method for estimating their reliability. As statistical inference requires large amounts of data measured under similar conditions in transcriptomics [11], the verification of network dynamics for known pathways must precede attempts to identify unknown network structures. It appears that each metabolic profile is measured under method-specific, presumably biased conditions.

Time-resolved target analysis is an effective way to observe biochemical dynamics. We systematically measured the level of 56 basic metabolites in rice leaves (*Oryza sativa *L. ssp. *japonica*) at hourly intervals over a 24-hr period. Our target and experimental conditions were strategically determined: 1) we focused on primary metabolic pathways consisting of carbon fixation/respiration- and nitrogen assimilation/dissimilation pathways, and comprehensively quantified related metabolites, 2) the photocycle was the sole environmental factor, and 3) measurements were made at 1-hr intervals to allow the observation of dynamic profiles.

High-throughput analysis was conducted with the capillary electrophoresis – mass spectrometry (CE-MS) technology we developed earlier [12-14], and has been applied to metabolic profiling in *Bacillus subtilis *extracts [15] and monitoring of genetic and environmental perturbations in *Escherichia coli *cells [16]. Each employed CE-MS method was able to detect charged low molecular metabolites in less than 30 min without requiring derivatization. Combined with diode array detection (CE-DAD), our technology is also applicable to quantifying small sugar compounds. We previously developed a sample preparation protocol that could extract metabolites with possibly minimal metabolic turnover [17]. By using the CE-MS and CE-DAD, we also succeeded in analyzing over eighty major metabolites (sugars, organic acids, amino acids, and nucleotides) in rice foliage. The current work is our first systematic time-course measurements of rice foliage throughout a day.

We applied four information-based methods to analyze the diurnal fluctuation of metabolites: 1) metabolic pathways were classified with SOM to monitor the metabolic dynamics in each time-step, 2) a phenotypic linkage map was constructed from the classified pathways by Sammon's 2D-network layout [18], 3) unidentified metabolites were predicted based on SOM analysis and chemical structures, and 4) rate-limiting enzymes were identified by hierarchical clustering on a correlation matrix. Here we show that combining metabolome analysis and information-based methods is an effective way to elucidate phenotypical metabolic network structures and underlying biological functions under diurnal rhythm fluctuations.

## Results

### Time-course data acquisition

**Figure 1:**
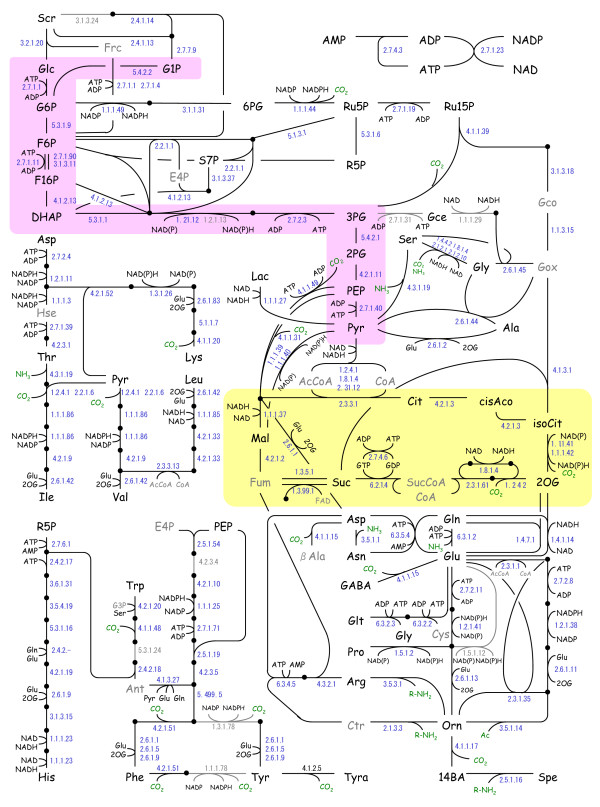
**Metabolic network of *oryza sativa *L. ssp. *Japonica***. Target metabolites and practical enzymatic reactions are shown. The number next to the line is the EC number. Colors indicate the ratio of metabolic levels in light and dark periods. Unidentified metabolites are gray and gray lines and EC numbers identify non-annotated enzymatic proteins. The red- and yellow shade show the glycolytic pathway and the TCA cycle respectively.

We extracted target metabolites existing in the primary metabolism such as the glycolytic pathway, the reductive- and oxidative pentose phosphate pathway, and the photorespiratory pathway, the tricarboxylic acid (TCA) cycle, and the amino acid biosynthetic pathway. Figure 1 presents the practical rice biochemical network that was constructed with our target metabolites based on annotated protein data from the KEGG pathway database [19], Swiss-Prot database [20], or Rice Annotation Project Data Base [21]. It shows the names of target metabolites and the EC number of enzymatic reactions; black dots are non-target metabolites. Although NH_3 _(also R-NH_2_) and CO_2 _were non-target compounds, they are shown in green to demonstrate in and out of carbon and nitrogen.

We selected eight enzymatic proteins that have not been annotated at this stage to determine whether they function in the rice plant. These enzymes and the judgment criteria are shown in Table 1. On the map, their EC numbers and lines are presented in gray.

**Table 1:** Selected non-annotated proteins expected to function in rice plant

EC Number	Enzyme name	Criterion for judgement	Ref.
1.1.1.29	hydroxypyruvate reductase; glycerate dehydrogenase	Enzymatic reduction of hydroxypyruvic acid to D-glyceric acid in higher plants, i.e. the leaves of pea, beet, tomato, radish, spinach, parsley, lettuce, corn, kohlrabi, and carrot.	[22]
		AK069655; Similar to 2-hydroxyacid dehydrogenase	RAP-DB*^1^
1.2.1.13	glyceraldehyde-3-phosphate dehydrogenase	AK071685; Similar to GADPH (383AA) (Fragment). AK67755; Similar to Glyceraldehyde-3-phosphate dehydrogenase (EC 1.2.1.13) (Fragment).	RAP-DB
1.3.1.78	arogenate dehydrogenase; prehenate dehydrogenase	TyrAAT1(AF434681) and TyrAAT2(AF434682) in *Arabidopsis thaliana *catalyze the oxidative decarboxylation of arogenate into Tyr in the presence of NADP. TyrAAT also exhibits prephenate dehydrogenase activity.	[23]
		Q5Z9H5_ORYSJ; Q5Z9H3_ORYSJ; Q5Z6Y1_ORYSJ, Putative arogenate dehydrogenase isoform 2	Swiss-Prot/TrEMBL*^2^
1.5.1.12	delta-1-pyrroline-5-carboxylate dehydrogenase	AK121765; Similar to delta-1-pyrroline-5-carbozylate dehydrogenase	RAP-DB
2.7.1.31	D-glycerate 3-kinase	GLYK family protein was purified and sequenced from *Arabidopsis thaliana*, identified as putative kinase-annotated single-copy gene At1g8038. This article suggests that an *Olyza sativa *PRK/UK-like protein, BAD73764, Os01g48990 is grouped with the GLYK kinase family.	[24]
3.1.3.24	sucrose-phosphatase	AK063330, AK071525, AK064563; Similar to sucrose-phosphatase	RAP-DB
4.2.3.4	3-dehydroquinate synthase	Pentafunctional aroma enzyme in Saccharomyces cervisiae includes EC 4.2.3.4, EC 4.2.1.10, EC 2.5.1.19, EC 1.1.1.25, and EC 2.7.1.71.	[25]
		AK071977; Similar to 3-dehydroquinate synthase-like protein (EC 4.2.3.4). Four other proteins were annotated.	RAP-DB
5.3.1.24	phosphoribosyl-anthranilate isomerase	J075072K08; Similar to phosphoribosylanthranilate isomerase	RAP-DB

*1: Rice Annotation Project Data Base [21]

*2: UniProt Knowledge base: Swiss-Plot and TrEMBL [20]

Sedoheptulose 1,7-bisphosphate (S17P) in the pentose phosphate pathway was not identified because the standard reagent was unavailable. Xylulose 5-phosphate (X5P) is a stereoisomer of Ribulose 5-phosphate (Ru5P) and their peak overlap in CE-MS analysis makes the identification even more difficult. Glyceraldehyde 3-phosphate (G3P) and oxaloacetate (OAA) were not accurately determined too, because they were readily reacted or decomposed.

The seventy selected target metabolites were classified into four groups according to their chemical structure-based physiochemical characteristics (Table 2). Group A contained amino acids and amines, group B organic acids and sugar phosphates, group C nucleotides and coenzymes, and group D sugars. Groups A, B, and C, consisting of ionic substances, were analyzed with three CE-MS methods for cationic, anionic, and nucleotide metabolites; analysis of group D was with a CE-DAD method. For CE separation, we used conventional sample preparation with simple and universal procedures without any derivatization process. As common preparation procedures were applicable under the four analytical conditions, we were able to determine simultaneously a wide variety of chemical compounds.

**Table 2:** The 70 target metabolites subjected to analysis of time-resolved dynamics and their abbreviation used in this article

Group A (CE-MS No.1)		Group B (CE-MS No.2)		Group C (CE-MS No.3)	
***Amino acids***		***Organic acids***		***Nucleotides***	
Ala	Alanine	*cis*Aco	*cis*-Aconitate	AMP	AMP
*β *Ala	*β*-Alanine	Cit	Citrate	ADP	ADP
GABA	*γ*-Aminobutyrate	*iso*Cit	*iso*-Citrate	ATP	ATP
Ant	Anthranilate	DHAP	Dihydroxyacetonephosphate	GDP	GDP
Arg	Arginine	Fum	Fumarate	GTP	GTP
Asn	Asparagine	Gce	Glycerate	***Coenzymes***	
Asp	Aspartate	Gco	Glycolate	NAD	NAD
Ctr	Citrulline	Gox	Glyoxylate	NADH	NADH
Cys	Cysteine	Lac	Lactate	NADP	NADP
Glu	Glutamate	Mal	Malate	NADPH	NADPH
Gln	Glutamine	2OG	2-Oxoglutarate	CoA	CoA
Glt	Glutathione red.	PEP	Phospho*enol*pyruvate	AcCoA	Acetyl-CoA
Gly	Glycine	6PG	6-Phosphogluconate	SucCoA	Succinyl-CoA
His	Histidine	2PG	2-Phosphoglycerate		
Hse	Homoserine	3PG	3-Phosphoglycerate		
Leu	Leucine	Pyr	Pyruvate	Group D (CE-DAD)	
				
Ile	*iso*-Leucine	Suc	Succinate	***Sugars***	
Lys	Lysine	***Sugar Phosphate***		Frc	Fructose
Orn	Ornithine	E4P	Erythrose 4-phosphate	Glu	Glucose
Phe	Phenylalanine	F16P	Fructose 1,6-bisphosphate	Suc	Sucrose
Pro	Proline	F6P	Fructose 6-phosphate		
Ser	Serine	G1P	Glucose 1-phosphate		
Thr	Threonine	G6P	Glucose 6-phosphate		
Trp	Tryptophan	R5P	Ribose 5-phosphate		
Tyr	Tyrosine	Ru15P	Ribulose 1,5-bisphosphate		
Val	Valine	Ru5P	Ribulose 5-phosphate		
***Amines***		S7P	Sedoheptulose 7-ohosphate		
14BA	1,4-Butanediamine				
Spe	Spermidine				
Tyra	Tyramine				

Plant seedlings were grown under a 13-hr light – 11-hr dark photocycle for 20 to 21 days. The level of the 56 metabolites was successfully quantified at hourly intervals over the course of 24 hr. We could identify the peak and determine the peak area for S7P but could not quantify its level, since the reagent was not available at the time of our CE-MS measurement; we later qualitatively identified its peak with the migration time ratio (MT/MT_IS_) of S7P to PIPES (internal standard). The other 13 metabolites were under the detection limit (signal-to-noise ratio (S/N) < 3); their names were colored gray in Figure 1.

**Figure 2:**
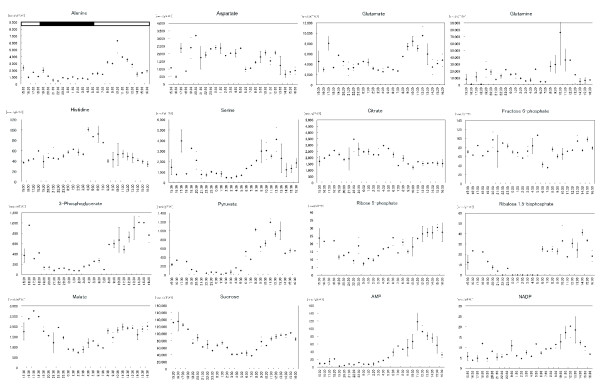
**Metabolic time-courses in rice foliage at the third-leaf stage**. Plantlets were grown under a 13-hr light – 11-hr dark photocycle. We applied 3 CE-MS methods and a CE-DAD method to analyze 69 major metabolites. Dynamic changes in the metabolite levels were assessed at hourly intervals over a 24 h period. Averages of 2 samples (± SEM) are shown. The top bar (shown in only Ala) indicates light and dark conditions.

In the course of 24 hr, the metabolites exhibited various fluctuations (Figure 2). Ru15P, the precursor of carbon fixation, manifested a variation synchronous with the photoperiod; its intracellular concentration increased under illumination and decreased in darkness. Several metabolites exhibited similar light-dependent variations in the reductive pentose phosphate pathway (3PG, R5P, and Ru5P), the glycolytic pathway (3PG, 2PG, PEP, Pyr), the TCA cycle (2OG, Suc, and Mal), and in sugars (Scr and Glc). Citrate, on the other hand, manifested opposite fluctuation changes. In the amino acid biosynthesis pathway, major amino acids (Ala, Asn, Gln, Glu, Gly, and Ser) accumulated during the light period. Minor amino acids that are synthesized from specific organic acids through several reaction steps (His, Ile, Leu, Lys, Phe, Trp and Val) accumulated during the dark period.

Table 3 shows the status of adenine nucleosides and nicotinamide coenzymes in the light and dark periods. Whereas the ratios of ADP, NADP, and NADH were almost equal in the light and dark periods, the ratios of AMP and NADPH were higher and those of ATP and NAD were lower in the light period (see Discussion).

**Table 3:** Status of adenine nucleosides and nicotinamide coenzymes in the light and dark period

	ATP AdN*^1^	ADP AdN	AMP AdN	NAD NiC*^2^	NADH NiC	NADP NiC	NADPH NiC
Light*^3^	0.21	0.40	0.40	0.36	0.10	0.09	0.44
Dark*^4^	0.45	0.43	0.11	0.55	0.09	0.05	0.31

*^1 ^AdN = ATP + ADP + AMP

*^2 ^NiC = NAD + NADH + NADP + NADPH

*^3 ^The average of all data throughout the light period

*^4 ^The average of all data throughout the dark period

### Self-organizing map and phenotypic linkage of metabolic modules

**Figure 3:**
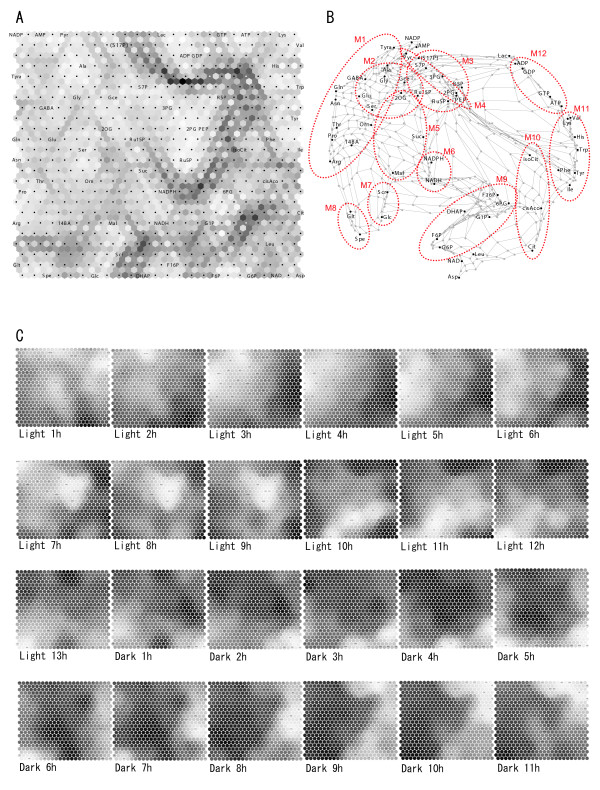
**Self-organizing map (SOM) Analysis**. **A**. U-matrix. Measured metabolites (n = 56) were arranged in a 20 × 20 lattice on the basis of diurnal change similarities. Light- and dark shading indicate high and low similarity, respectively. **B**. Phenotypic linkage map (PLM). The linkage among metabolites based on dynamic similarity is expressed as the distance on the quadratic plane. The metabolites were assigned to 14 metabolic modules that fluctuated synchronously; most contained traditional metabolic pathway networks or similar compounds. M1, major amino acid; M2, related to photorespiratory pathway intermediates; M3, pentose phosphate pathway; M4, latter half of the glycolytic pathway; M5, latter half of the TCA cycle; M6, environmental stress response; M7, sugars; M8, NADH and NADPH; M9, first half of the glycolytic pathway; M10; first half of the TCA cycle; M11, minor amino acids; M12, nucleoside tri- and diphosphates. **C**. Time-resolved layout. The relative levels of metabolites are shown for every time point from the start of the light period to the end of the dark period. Light and dark shading indicate high and low levels.

To visualize the functioning networks throughout a 24-hr period, we classified the metabolites according to similarities in their time-dependent behavior by using Kohonen's self-organizing map (SOM) and Sammon's 2D-network layout (Sammon map). The time-dependent levels of each metabolite were represented as a 24-dimensional vector. On the SOM, the 57 metabolites were classified into a 24 × 24 lattice on the basis of vector similarity. The map was roughly divided into two major groups (see the dark gray line in Figure 3A). Metabolites with high levels in the light period are in the left area; those with high levels in the dark period are on the right in the map. On the SOM, each group was further classified and assigned to subgroups consisting of nitrogen- and carbon-assimilating compounds. Certain amino acids were arranged near their precursor organic acids, e.g., Glu/2OG. Gly, Ser, and Ala were grouped with synthetic pathway intermediates such as Pyr and Gce. The degree of similarity among metabolites was quantitatively visualized on the Sammon map; it shows approximate distances between metabolites on the SOM according to the Euclidean distance of the input vectors (Figure 3B). When we merged neighboring metabolites on the Sammon map we obtained 12 subsets of metabolites. Each subset is composed of metabolites that exhibit synchronous, time-dependent fluctuations, a "metabolic module". Metabolites in the same module were often neighbors in a traditional metabolic pathway network. Products that accumulated during the light period were arranged in subsets M1 – M8. They included the module for the reductive pentose phosphate pathway (M3), the photorespiratory pathway (M2), the latter half of the glycolytic pathway (M4), the latter half of the TCA cycle (M5), sugars (M7), and major amino acids (M1). Also included in this group were NADPH and NADH (M6), glutathione and spermidine (M8). Subsets M9 – M12 included the first half of the glycolytic pathway (M9), the first half of the TCA cycle (M10), and minor amino acids (M11); also included were the nucleoside tri- and diphosphates (M12). Thus, our SOM analysis correctly reflected the phenotypic metabolic variations that indicate functioning biochemical pathways, and therefore represents a phenotypic linkage map (PLM).

The advantages of this analysis became even more apparent upon time-resolved analysis of metabolite levels (Figure 3C), which allowed visualization of the dynamic activity of these metabolic modules (see Discussion).

## Discussion

### Estimation of unidentified metabolites with SOM analysis

Although S17P could not be directly identified, we hypothesized that its peak could be identified in CE-MS data by combining SOM analysis with knowledge of the chemical structure. We identified a candidate peak among several peaks on selected ion electropherograms using a simple estimation method. As electrophoretic mobility is proportional to the ionic charge of the solute and inversely proportional to the size of the ionic molecule related to the hydrated ionic radius of a spherical molecule [26], we used the cubic root of the molecular weight as a substitute parameter for the radius. Indeed, the cubic root of molecular weights of 3 metabolites of similar chemical structure, Ru5P, F6P and S7P, were linearly correlated with migration time ratios (r > 0.999), when PIPES was used as an internal standard (Table 4).

**Table 4:** Estimated migration-time of unidentifiable metabolites based on the molecular weight of similar metabolites

Compound	Formula	M.W.	M.W.^1/3^	MT/MT_IS_
Ru5P	CH_2_(OH)CO [CH(OH)]_2_CH_2_OPO_3_H_2_	230.0192	6.127	1.029
F6P	CH_2_(OH)CO [CH(OH)]_3_CH_2_OPO_3_H_2_	260.0298	6.383	1.080
S7P	CH_2_(OH)CO [CH(OH)]_4_CH_2_OPO_3_H_2_	290.0403	6.619	1.125
Ru15P	CH_2_(OPO_3_H_2_)CO [CH(OH)]_2_CH_2_OPO_3_H_2_	309.9854	6.768	0.847
F16P	CH_2_(OPO_3_H_2_)CO [CH(OH)]_3_CH_2_OPO_3_H_2_	339.9960	6.980	0.895
S17P	CH_2_(OPO_3_H_2_)CO [CH(OH)]_4_CH_2_OPO_3_H_2_	370.0065	7.179	0.941*

*Estimated value. MT/MT_IS _was calculated by linear approximation

The estimate for S17P was performed using linear approximation with Ru15P and F16P. The estimated migration time ratio (MT/MT_IS_) of S17P was 0.941 (Table 4). Several peaks were observed at a mass-to-charge ratio (m/z) of 369. A peak of MT/MT_IS _= 0.909 (m/z = 369) was identified within ± 5.0% of the predicted values.

Next, the absence of other metabolites with similar chemical structures was verified with the KEGG ligand database [27]. Note that except for S17P, metabolites were cyclic or non-anionic compounds.

Finally, we obtained the normalized time-course of the putative S17P by calculating the ratio of the peak area of putative S17P to PIPES. Integration of these data into the SOM analysis showed that this putative S17P marker was near metabolites in the reductive pentose phosphate pathway (Figure 3A) or the metabolic module M3 in PLM.

Unfortunately, the above result includes some speculation; most peaks of putative S17P were below the detection limits (S/N < 3) and the peak was not detected in the dark period. In the SOM analysis, the peak area of such undetected metabolite was calculated as zero. Nevertheless, the proposed estimation method seems to be effective in identifying unknown metabolites.

### Detection of metabolic bottlenecks by pair-wise correlation analysis

In previous studies, Peason's correlation coefficients of metabolite pairs (pair-wise correlation) were applied to construct a metabolic correlation network [5,10,28]. A correlation coefficient is an index of co-linearity between two variables. If two metabolites, A and B, are always equilibrated, i.e., [A]/[B] = K_eq _(constant), then their relationship is linear and shows a high correlation. Although real metabolic pathways are dynamic and constantly regulated by their influx and/or efflux, the pathway components that are blocked by rate-limiting enzymes should exhibit approximate linearity. For example, 3PG, 2PG, and PEP in the glycolytic pathway are positioned between two rate-limiting enzymes, phosphoglycerate kinase (EC 2.7.2.3) and pyruvate kinase (PK; EC 2.7.1.40), both of which are regulated by the ATP/ADP ratio (Figure 1). The correlation coefficients among these three metabolites throughout a 24-hr period were over 0.90, whereas the correlation coefficient between PEP and Pyr, limited by PK, was under 0.50. Thus, pair-wise correlation analysis is effective for the identification of metabolic modules that are regulated by rate-limiting enzymes.

**Figure 4:**
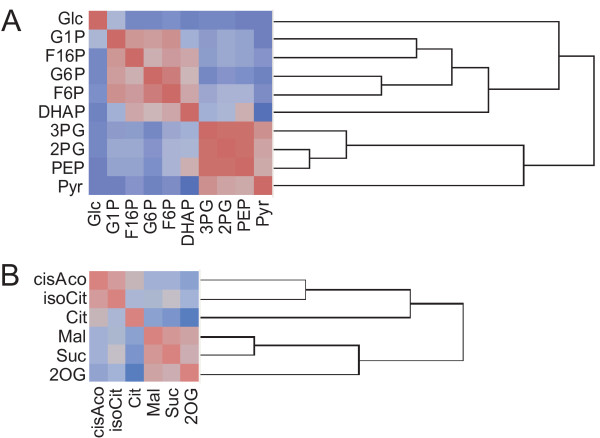
**Hierarchical cluster analysis**. **A**. Cluster analysis (Ward's method [26]) was applied to the correlation matrix composed of metabolic intermediates in the glycolytic pathway. The generated dendrogram was clustered into regulatory units by the ATP/ADP ratio; hexokinase (EC 2.7.1.1), phosphoglycerate kinase (EC 2.7.2.3), and pyruvate kinase (EC 2.7.1.40). **B**. As well as in the TCA cycle, the dendrogram was divided into two major groups at the rate-limiting steps; citrate synthase (CS; EC 2.3.3.1), and NADP-dependent isocitrate dehydrogenase (ICDH; EC 1.1.1.42).

We used a hierarchical clustering algorithm, Ward's method [29], to classify metabolites in the glycolytic pathway (Figure 1) on the basis of their correlation matrix that was computed using all data throughout the 24-hr period. Indeed, a dendrogram identified the steps regulated by the ATP/ADP ratio (Figure 4A). On the other hand, it did not identify phosphofructokinase I (PFK-1; EC 2.7.1.11) as a rate-limiting enzyme. Although it is regulated by the ATP/ADP ratio in animal cells, another enzyme, pyrophosphate fructose 6-phosphate 1-phosphotransferase (EC 2.7.1.90), seems to be active in plant cells and may be independent of the ATP/ADP ratio [30].

The same cluster analysis was also applied to the TCA cycle intermediates (Figure 1), and the dendrogram revealed the rate-limiting enzymes in the cycle again (Figure 4B): citrate synthase (CS; EC 2.3.3.1), and NADP-dependent isocitrate dehydrogenase (ICDH; EC 1.1.1.42). This suggests that the classification of metabolites along enzymatic steps can help to reveal bottleneck enzymes.

### Time-resolved carbon/nitrogen metabolomics

Inspection of the time-course of metabolic modules allowed us to better understand the carbon and nitrogen (C/N) assimilation/dissimilation process and their underlying function during a 24-hr period (Figure 3C).

In the first half of the light period, some accumulation emerged for carbon-fixed products: Pyr, 2OG, and photorespiratory pathway intermediates (metabolic module M2). This coincides with carbon fixation by activation of several light-dependent enzymes including rubisco (EC 4.1.1.39) at the start of light exposure [31], as shown by the accumulation of Ru15P, Gce and triose derivatives at the beginning of the light period (light 1 – 3 hr). The slow accumulation was partly attributable to the very slow metabolic turnover of rubisco [32]. Likewise, major amino acids and amines including Glu and Gln, the source compounds of nitrogen assimilation as amino-group acceptor/donor [33,34], also accumulated in the first half of the light period (M1). This coincides with the diurnal metabolic dynamics and the activities of key enzymes in tobacco plant [35]. For example, NR activity is known to remarkably increase immediately after the start of light exposure and decrease at midday.

On the other hand, the glycolytic pathway and the reductive pentose phosphate pathway intermediates reached their highest levels (M3, M4) at midday, and sugars peaked at the end of the light period (M7).

We can hypothesize that carbon fixed in the first half of the light period moves down the glycolytic pathway and the TCA cycle, and amino acid biosynthesis progresses using generated Glu, Pyr, and 2OG. In the latter half of the light period, the flow of fixed carbon leads to the accumulation of the intermediates in the pentose phosphate pathway and to sucrose synthesis by inhibiting the production of ammonia, Pyr, and 2OG.

From the end of the light period through the first half of the dark period, we noted an increase in sugar phosphates from the first half of the glycolytic pathway (metabolic module M9). Around midnight, the accumulation of a few organic acids in the first half of the TCA cycle (metabolic module M10) was observed, suggesting the activation of the TCA cycle.

In the latter half of the dark period, the level of minor amino acids was increased (metabolic module M11), although they are synthesized from diverse biochemical pathways. The good correlation among these minor amino acids, also reported in potato and wheat [36], is attributable to the fact that the ratio between Gln and 2OG regulate minor amino acids in bacteria and fungi through the reaction Glu + 2-oxo acid ↔ amino acid + 2OG [37]. Under our experimental conditions, the Glu/2OG ratio was much higher in the dark- than in the light period (22.9 vs. 7.2) and the amino group can easily transferred to 2-oxo acids to produce amino acids.

### Adenine nucleoside and nicotinamide coenzyme status

ATP and ADP were placed in the dark-activated group in PLM (metabolic module M12); they were accumulated at the end of the dark period, and decreased by illumination (Figure 3C). On the other hand, AMP was placed in the light-activated group peaking at midday. The reason for fluctuations of adenylate is unknown. Previous observations also do not coincide in the adenylate levels during the light- and dark period. In sugar beet leaves, all adenylate levels increased in the light period [38]. In spinach leaves and wheat leaf protoplast, ATP increased but ADP and AMP decreased under light [39,40]. In Crassulacean-acid metabolism (CAM) species, on the contrary, ATP decreased but ADP and AMP increased [41]. Such differences may result from different dynamics in cytosol, chloroplasts, and mitochondria [40].

We extrapolate that the lower ATP ratio during the light period was caused by an excess demand of ATP by intra- and extra cellular processes for carbon fixation and nitrogen assimilation against ATP supply from photosynthesis. In theory, the amount of ATP consumption in the reductive pentose phosphate pathway and the photorespiratory pathway is more than ATP production in the photophosphorylation [42]. Beside this, nitrogen assimilation process, intracellular transport of the assimilation products, and sucrose synthesis and its translocation are also accompanied by ATP. Therefore the dark respiration makes a considerable contribution to produce ATP even in the light. However, granted that ATP supply is insufficient in the light, high metabolic turnover of adenylate kinase (EC 2.7.4.3) would immediately work to reproduce ATP from ADP that leads to increase of AMP. Further investigation is necessary to clarify the adenylate dynamics among cell compartments.

In our analysis, NADPH and NADH behaved similarly (metabolic module M6), whereas NADP and NAD did not. As NADPH and NADH were respectively generated by their unique reaction of reducing NADP and NAD, dependence on the intracellular oxidation-reduction state shifted the formation of oxidation and reduction. In PLM, however, NADP was placed in the light-activated- and NAD in the dark-activated group. This suggests that highly concentrated NAD in the dark is converted to NADPH via NADP in the light period. It was reported that the NADPH/NAD ratio is the inverse of the ATP/ADP ratio in guard cell protoplast, which indicates that ATP phosphorylates NAD in the light period by NAD kinase (EC 2.7.1.23) and the generated NADP is reduced to NADPH in the course of photosynthesis [43].

The ratios of NADH to NAD and NADPH to NADP were 0.16–0.29 and 6.2–6.6. The observed difference in the tendency of oxidized- or reduced form indicates their different cellular roles. NADH is used for oxidative phosphorylation, and a low NADH/NAD ratio constrains this process. On the other hand, NADPH is used for the reductive biosynthesis of metabolites, and the high ratio of NADPH/NADP favors the reduction of metabolites.

### Environmental stress response

It is remarkable that Glt (GSH; gamma-glutamylcysteinyl glycine) and Spe exhibited similar fluctuation patterns (metabolic module M8). Both peaked at the end of the light period and again just after midnight, suggesting the existence of common regulatory factors. GSH plays a central role in the antioxidant defense by eliminating harmful peroxide during photosynthesis and oxidative phosphorylation [44]. Polyamines, including spermidine, are also effective antioxidants under various environmental stress conditions [45]. During photosynthesis, GSH is converted to oxidized dithiol (GSSH) to eliminate oxidative stress, and upon the reduction of NADPH, GSSH can be converted back to GSH by glutathione reductase (GR; EC 1.8.1.7, annotated in rice plant). Our finding that NADPH reached its highest level at a few hours before the end of the light period is consistent with the above observation (Figure 3C), although the connection remains speculative. The relative contribution of NADPH and NADH to the generation of GSH and spermidine requires further investigation.

## Conclusion

We intended to analyze the rice plant metabolism and to reconstruct its phenotypic networks in an effort to explain underlying biological functions. Our CE-MS technology provided a comprehensive high-throughput system with easy sample preparation and facilitated the generation of high-resolution metabolic time-courses. Data mining with statistical techniques and SOM analysis revealed synchronous dynamics in metabolic modules downstream of C and N assimilation and dissimilation processes and stress responses. Our system was able to discriminate unidentified metabolites and identify bottleneck enzymatic steps. In a comprehensive approach such heuristics become increasingly important because with current technology, the determination of all network components is virtually impossible. For a more precise investigation of biochemical networks, expansion of target metabolites and determination of metabolite levels in each cellular compartment may be suggested. There are technical hurdles, however, in separating organelles without disturbing a wide range of metabolites inside them. Without much technical advancement, therefore, it seems difficult to repeat our time-course measurement for any single cellular compartment although there are reports for such a challenge [46]. Finally, for the analysis part, it is necessary to couple biological information with computer simulations based on large-scale time-resolved measurements of metabolites, proteins, and mRNAs.

## Methods

### Plant materials

Young seedlings of rice plants, *Oryza sativa *L. ssp. *japonica *Haenuki, at the third-leaf stage were cultured as follows. Rice seeds were germinated on filter paper soaked with Milli-Q water and kept at 30°C in a dark room for 2 days. After germination, the plantlets were placed on rock fiber (35 × 35 × 40 mm; Nittobo, Tokyo, Japan), and grown in a growth chamber (FLI-301N, Tokyo Rika Kikai, Tokyo, Japan) for 18 days. The temperature and light conditions were 25°C and 365 μE·m^-2^s^-1 ^for 9 hr (light), 20°C and 0 μE·m^-2^s^-1 ^for 11 hr (dark), and 150 μE·m^-2^s^-1 ^for 2 hr between light and dark. The plants were watered with Kasugai water culture solution (18.9 mg/L (NH_4_)_2_SO_4_, 10.1 mg/L Na_2_HPO_4_·12H_2_O, 4.7 mg/L KCl, 0.79 mg/L CaCl_2_, 3.0 mg/L MgCl_2_, 0.17 mg/L·FeCl_3_·6H_2_O, and HCl to adjust the pH to 5.0 – 5.5) [47].

### Reagents

Piperazine-1,4-bis(2-ethanesulfonic acid) (PIPES) was purchased from Dojindo (Kumamoto, Japan), methionine sulphone from Avocado Research (Heysham, Lancashire, UK). All other reagents were obtained from conventional commercial sources. Individual stock solutions, at a concentration of 10 or 100 mM, were prepared in Milli-Q water, 0.1 N HCl, or 0.1 N NaOH. The working standard mixture was prepared by diluting these stock solutions with Milli-Q water just before injection. All chemicals used were of analytical or reagent grade. Water was purified with a Milli-Q purification system (Millipore, Bedford, MA, USA).

### Sample preparation

Leaves were harvested (fresh weight approximately 100 mg (6 seedlings)) and frozen in liquid nitrogen to stop enzymatic activity. They were mashed in a Multi-Beads Shocker (Yasuikikai, Osaka, Japan) at 2000 rpm for 10 sec and 0.5 mL of ice-cooled methanol, including 400 μM PIPES and methionine sulphone as an internal standard, was added to dissolve phospholipid membranes and inactive enzymes. Then 0.5 mL ice-cold Milli-Q water was added and the sample was ultrafiltered through a 5-kDa cut-off filter at 9058 g for 10 min to remove proteins, phospholipids, chlorophyll, and other high-molecular-weight impurities. The filtrate was analyzed by CE-MS and CE-DAD methods. To obtain sufficient sensitivity for the analysis of nucleotides, coenzymes, and sugars, the filtrate was concentrated 5-fold by lyophilization [17].

### Instruments

All CE-MS experiments were performed by Agilent CE capillary electrophoresis. We used a 1100 series MSD mass spectrometer, a 1100 series isocratic HPLC pump, a G1603A CE-MS adapter kit, and a G1607A CE-ESI-MS sprayer kit (Agilent Technologies). CE-DAD experiments were performed by Agilent CE capillary electrophoresis with a built-in diode-array detector. G2201AA Agilent ChemStation software for CE was used for system control, data acquisition and analysis, and MSD data evaluation.

### Analytical conditions

The compounds were analyzed in four groups using three CE-MS methods and one CE-DAD method.

a) Cationic metabolites (amino acids and amines) were analyzed with a fused-silica capillary (50 μm i.d. × 100 cm total length), with 1 M formic acid as the electrolyte. The sample was injected at an injection pressure of 5.0 kPa for 3 sec (approximately 3 nL). The applied voltage was set at 30 kV. The capillary temperature was set to 20°C, and the sample tray was cooled to below 5°C. The sheath liquid (5 mM ammonium acetate in 50% [v/v] methanol-water) was delivered at 10 μL/min. ESI-MS was conducted in positive ion mode; the capillary voltage was set at 4000 V. A flow rate of heated dry nitrogen gas (heater temperature 300°C) was maintained at 10 L/min [12].

b) Anionic metabolites (organic acids and sugar phosphates) were analyzed with a cationic polymer-coated SMILE(+) capillary (Nakalai Tesque, Kyoto, Japan). The electrolyte for CE separation was a 50 mM ammonium acetate solution (pH 8.5). The sample was injected at an injection pressure of 5.0 kPa for 30 sec (approximately 30 nL). The applied voltage was set at -30 kV, and the capillary temperature was set to 30°C. ESI-MS was conducted in negative ion mode; the capillary voltage was set at 3500 V. Other conditions were as in the cationic metabolite analysis [13].

c) Nucleotides and coenzymes were analyzed with an uncharged polymer-coated gas chromatograph capillary, polydimethylsiloxane (DB-1) (Agilent Technologies). The electrolyte for CE separation was 50 mM ammonium acetate solution (pH 7.5). The applied voltage was set at -30 kV and a pressure of 5.0 kPa was added to the inlet capillary during the run. Other conditions were as in the anion analysis [14].

d) Sugars were analyzed with a fused-silica capillary (50 μm i.d. × 112.5 cm total length, 104 cm effective length). Basic anion buffer for CE (Agilent Technologies) was the electrolyte. The sample was injected at a pressure of 5.0 kPa for 10 sec (approximately 10 nL). The applied voltage was set at -25 kV; the capillary temperature, regulated with a thermostat, was 25°C. Sugars were detected by indirect UV detection using a diode-array detector. The signal wavelength was set at 350 nm with a reference at 230 nm [48].

### Self-organizing map (SOM) analysis

A free software package, SOM -PAK [49], was used to compute both the SOM and the Sammon map. Before SOM analysis, the observed time-course data for 58 metabolites (including an estimate of S17P) were smoothed by averaging the adjacent data points using a sliding window of width 3, to reduce high-frequency noise presumably originating from individual differences in plant seedlings, rapid oscillations in metabolism, or measurement errors. The missing data points were extrapolated by linear approximation between prior and subsequent data values. Among the 57 metabolites evaluated at 26 time points, only 30 data points could be extrapolated due to the detection limit or contamination of other unidentifiable peaks. The SOM is a map from the input *n*-dimensional data space (input layer) to a two-dimensional array of nodes (output layer). The vectors in the output layer are the parametric reference vector ***m***_***i***_, which has *n *elements. An input data vector, ***x***, is compared with ***m***_***i***_, and the best-match vector, which is the smallest Euclidean distance |*x *- ***m***_***i***_|, is mapped onto this location. During learning, nodes that are topographically close in the array up to a certain distance activate each other to learn from the same input vector, and the reference vectors are corrected so that they become close to the input vector. Thus,

***m***_***i***_(t + 1) = ***m***_***i***_(t) + *h*_*ci*_(t) [*x*(t) - ***m***_***i***_(t)],

where t is an integer, the discrete-time coordinate, and *h*_*ci*_(t) is the neighborhood kernel, a function defined over the lattice points. The neighborhood size, *N*_*c*_, around node *c *is a function of time, and *h*_*ci *_is defined as

$$\begin{array}{ll} h_{ci}=\alpha (\text{t}) & \left(i\in N_{c}\right)\\ h_{ci}=0 & (i\notin N_{c}), \end{array}$$

where *α*(t) is a monotonic decreasing function of time (0 <*α*(t) < 1) called the "learning rate". The learning rate function was defined as

*α*(*t*) = *α*(0)(1.0 - *t*/*T*),

where *α*(0) is the initial learning rate and *T *the running length (number of steps) in training. In this study, 58 metabolic time-courses were formatted and classified in a 24 × 24 hexagonal lattice. The applied SOM parameters were: initial radius of the training area = 12, initial learning rate = 0.025, running length = 65 000.

### Metabolic pair-wise correlation

Significance levels for Pearson correlation coefficient *r *were computed depending on the number of metabolite pairs *n *found throughout the light and dark period, respectively, by calculating t-scores given by *t *= *r *(*n *- 2)^0.5^/(1 - *r*)^0.5^. The critical t-score was set to correspond to the commonly used p-value of 0.05 in two-sided tests.

### Hierarchical clustering

Among several algorithms for clustering analysis, we chose Ward's method [29] in JMP software (ver. 6.0.0; SAS Institute Inc. Cary, NC). Starting from trivial clusters each containing one object only, Ward's method iteratively merges two clusters that will result in the smallest increase in the sum of the square of their differences (i.e., variance). At each step, all possible mergers of two clusters are tried and their variance is computed. The difference between clusters is calculated by the equation:

$$d(a,b)=\frac{n_{a}n_{b}}{n_{a}+n_{b}}{\left(x_{a}-x_{b}\right)}^{2}$$

## Authors' contributions

SS conceived this study, performed the biochemical- and the computational experiments, and wrote the manuscript. MA provided intellectual help for the computational analysis and together wrote the manuscript. TN advised the experimental design. TS and MT supervised the research. All authors read and approved the final manuscript.
